# The Rate of NF-κB Nuclear Translocation Is Regulated by PKA and A Kinase Interacting Protein 1

**DOI:** 10.1371/journal.pone.0018713

**Published:** 2011-04-27

**Authors:** Charles C. King, Mira Sastri, Philip Chang, Juniper Pennypacker, Susan S. Taylor

**Affiliations:** 1 Department of Pediatrics, University of California San Diego, La Jolla, California, United States of America; 2 Department of Pharmacology, University of California San Diego, La Jolla, California, United States of America; 3 Department of Chemistry and Biochemistry, University of California San Diego, La Jolla, California, United States of America; 4 The Howard Hughes Medical Institute, University of California San Diego, La Jolla, California, United States of America; Emory University, United States of America

## Abstract

The mechanism of PKAc-dependent NF-κB activation and subsequent translocation into the nucleus is not well defined. Previously, we showed that A kinase interacting protein 1 (AKIP1) was important for binding and retaining PKAc in the nucleus. Since then, other groups have demonstrated that AKIP1 binds the p65 subunit of NF-κB and regulates its transcriptional activity through the phosphorylation at Ser 276 by PKAc. However, little is known about the formation and activation of the PKAc/AKIP1/p65 complex and the rate at which it enters the nucleus. Initially, we found that the AKIP1 isoform (AKIP 1A) simultaneously binds PKAc and p65 in resting and serum starved cells. Using peptide arrays, we refined the region of AKIP 1A binding on PKAc and mapped the non-overlapping regions on AKIP 1A where PKAc and p65 bind. A peptide to the amino-terminus of PKAc (CAT 1-29) was generated to specifically disrupt the interaction between AKIP 1A and PKAc to study nuclear import of the complex. The rate of p65 nuclear translocation was monitored in the presence or absence of overexpressed AKIP 1A and/or (CAT 1-29). Enhanced nuclear translocation of p65 was observed in the presence of overexpressed AKIP1 and/or CAT 1-29 in cells stimulated with TNFα, and this correlated with decreased phosphorylation of serine 276. To determine whether PKAc phosphorylation of p65 in the cytosol regulated nuclear translocation, serine 276 was mutated to alanine or aspartic acid. Accelerated nuclear accumulation of p65 was observed in the alanine mutant, while the aspartic acid mutation displayed slowed nuclear translocation kinetics. In addition, enhanced nuclear translocation of p65 was observed when PKAc was knocked-down by siRNA. Taken together, these results suggest that AKIP 1A acts to scaffold PKAc to NF-κB in the cytosol by protecting the phosphorylation site and thereby regulating the rate of nuclear translocation of p65.

## Introduction

Translocation of the ubiquitous cAMP-dependent protein kinase (protein kinase A; PKA) to specific sites in cells helps elicit selective responses to biological inputs from external stimuli [1], [2], [3]. Targeting and compartmentalization of PKA has been mostly thought to be mediated by A-kinase-anchoring proteins (AKAPs) that bind the dimerization/docking domain of the regulatory subunits and scaffold PKA to substrates [4], [5], [6], [7], [8], [9]. Activation of the PKA catalytic subunit (PKAc) following cAMP stimulation, results in from the regulatory subunits and phosphorylation of substrates proximal to or tethered at AKAPs. Catalytically active PKAc also translocates to the nucleus where it phosphoylates the transcription factor cAMP response element binding protein (CREB) to regulate gene expression [10]. A Kinase Interacting Protein (AKIP1) was identified as a novel PKAc binding protein that targets PKAc to specific locations within cells [11]. AKIP1 binds the amino terminal tail of PKAc (N-Tail: residues 1-39). The N-Tail is a genetically diverse region in the protein that precedes the conserved core and acts to target the protein to distinct subcellular locations through myristylation, phosphorylation, and deamidation [12], [13], [14], [15], [16]. One of the functions of AKIP1 appears to be retention of PKAc in the nucleus of cells [11].

PKAc has also been shown to be associated with the NF-κB:IκB complex. NF-κB is a transcription factor that induces the expression of genes involved in many biological responses including inflammation, cell proliferation, and survival [17], [18], [19], [20], [21]. The majority of NF-κB exists as a heterodimer of p65/p50 proteins sequestered in the cytosol and bound to inhibitory IκB proteins [22]. Stimulation of cells with cytokines, including TNFα, activate IκB kinases that phosphorylate IκB and result in ubiquitination and proteosomal degradation of the protein [23], [24], [25]. The free p65/p50 complex enters the nucleus and initiates transcription of downstream effector genes [26], [27]. Identification of cytosolic complexes containing NF-κB, IκB, and PKAc have previously demonstrated that cAMP independent signaling may play a regulatory role in NF-κB mediated nuclear translocation and transcription [28]. Phosphorylation by PKAc was mapped to serine 276 in the p65 subunit of NF-κB, and showed pleiotropic effects including recruitment of CREB binding protein/p300 and subsequent acetlyation, as well as displacement of histone deacetylase [29], [30], [31]. The importance of this site has recently been studied through mutational analysis. Mutation of serine 276 to alanine is embryonic lethal despite translocation to the nucleus and interaction with DNA, demonstrating the regulatory importance of this site [32].

Two recent studies identified AKIP1 as a p65 binding protein [33], [34]. In the first study, the 1B isoform of AKIP1, upon neddylation, was found to bind p65, and recruit histone deacetylase, SirT1, to the complex resulting in transcriptional repression [33]. The second study identified p65 as an AKIP 1A binding partner through a yeast two-hybrid screen. AKIP 1A was recruited to a nuclear complex that contained p65 and PKAc. The net result of this complex was an increase in NF-κB dependent transcription.

In this study, we wanted to examine the effect of PKAc and AKIP1 on the rate of translocation of NF-κB into the nucleus. We provide evidence that AKIP1 acts as a molecular scaffold that simultaneously binds PKAc and p65 in the cytosol and disruption of this complex alters the rate at which NF-κB enters the nucleus. Our data also suggests that phosphorylation of p65 by PKAc is abrogated by AKIP1 in the cytosol and this allows p65 to translocate faster into the nucleus.

## Results

### AKIP1 binds to PKAc and enhances its transcriptional activity upon stimulation

**Figure 1:**
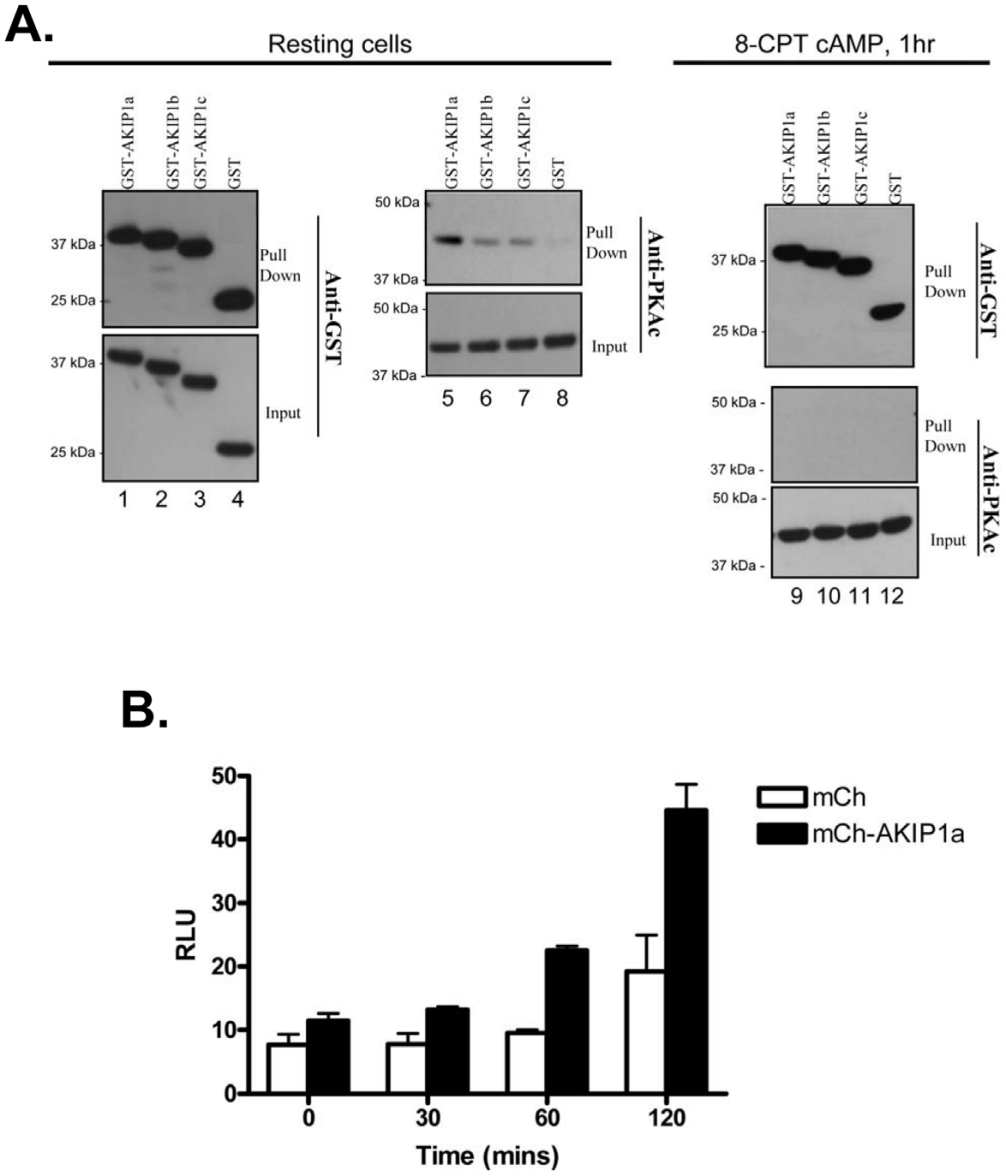
The AKIP 1A isoform binds to endogenous PKAc and enhances transcription. A) Western blots of detergent soluble lysates from HEK 293 cells alone or incubated with 8-CPT-cAMP (50 µM) showing immunoprecipitated and total protein levels for transfected GST AKIP constructs and endogenous PKAc. Lysates containing GST- AKIP 1A, AKIP 1B, AKIP 1C, and GST alone were incubated with GST resin (GE Healthcare) for 1 h, washed, and analyzed on 10% SDS-PAGE and western blotted with anti-GST antibody (lanes 1–4), anti-PKAc antibody (lanes 5–8), or both (lanes 9–12). B) HeLa cells were transfected with the cAMP response element upstream of the luciferase reporter in the presence of either mCherry-AKIP 1A or mCherry alone. Cells were treated with forskolin for up to 2 hours and PKAc dependent transcription was measured.

Previous work identified AKIP1 as a PKA binding protein that regulates nuclear localization of the catalytic subunit of PKA (PKAc) (11). Three AKIP1 isoforms have been identified, but no information exists on how these different isoforms bind PKAc in resting and stimulated cells. In initial studies, the ability of each AKIP1 isoform to bind endogenous PKAc was evaluated (Fig. 1*A*). GST-tagged AKIP 1A, AKIP 1B, and AKIP 1C bound PKAc in resting HEK 293 cells (lanes 5–7), but not GST alone (lane 8). Each AKIP1 isoform, as well as GST alone, expressed and bound to beads equally (lanes 1–4). Since there was a preference for PKAc to bind AKIP 1A stronger than other AKIP1 isoforms (lane 5), this isoform was used throughout this study. Treatment of HEK 293 cells with 8-CPT-cAMP for 1 hour to activate PKAc disrupted the interaction of PKAc with all three AKIP1 isoforms.

To further confirm that overexpression of AKIP1 facilitates translocation of PKAc into the nucleus, PKAc-mediated transcription was explored. Previously, HeLa cells overexpressing AKIP1 demonstrated enhanced nuclear accumulation of endogenous PKAc, and this effect could be blocked by disruption of the AKIP1-PKAc interaction. To determine whether the observed enhanced PKAc nuclear translocation resulted in a functional change, HeLa cells were co-transfected with mCherry-AKIP 1A and a luciferase gene reporter downstream of a cAMP response element (Fig. 1B) and stimulated with forskolin (to increase intracellular cAMP levels) and IBMX (a phosphodiesterase inhibitor) for 0, 30, 60 and 120 minutes. Upon activation, enhanced luciferase activity was observed in cells expressing mCherry AKIP 1A, suggesting that AKIP1 acts to shuttle PKAc into the nucleus and subsequently dissociate from it.

### Formation of a multiprotein complex containing AKIP 1A, PKAc, and p65

**Figure 2 pone-0018713-g002:**
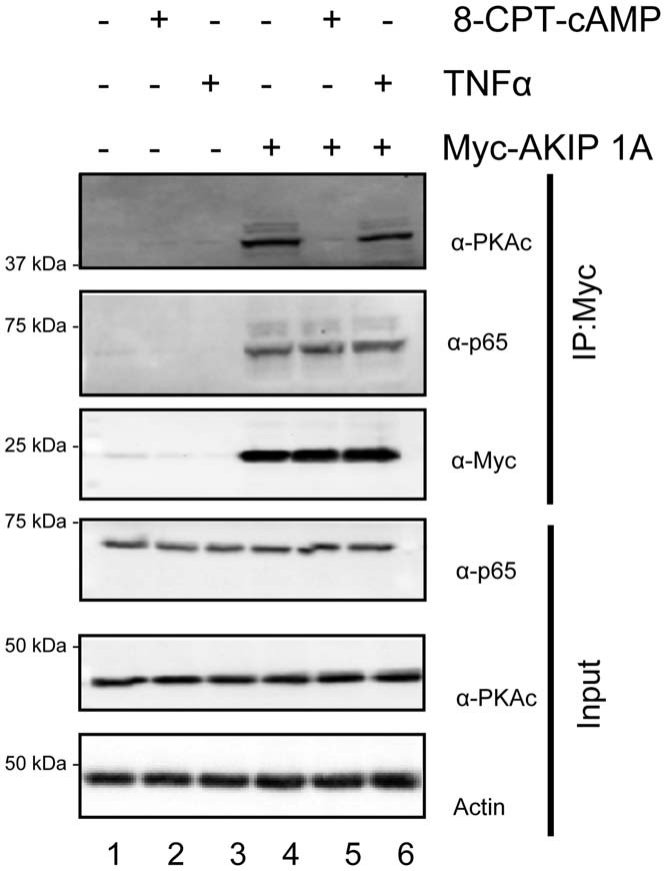
Interaction of PKAc with AKIP 1A is stimulus dependent, but the interaction of p65 remains constant. HEK 293 cells were transiently transfected with cDNA for wild-type Myc-AKIP 1A (lanes 4–6) or control vector (lanes 1–3). Cells were stimulated with 8-CPT-cAMP (50 µM; lanes 2 and 5) or TNFα (1 ng/ml; lanes 3 and 6) for 60 min and probed for endogenous PKAc, p65, and actin, as well as Myc by Western blot.

PKAc and the p65 subunit of NF-κB were identified from two independent yeast two-hybrid screens as AKIP1 binding proteins. In the presence of phorbol esters, Gao *et al.* observed a complex of C subunit, AKIP 1A and p65 and hypothesized that this complex forms in the nucleus (34). To extend these observations, we first wanted to determine whether this complex was present in whole cell lysates from serum starved cells, or in cells stimulated with 8-CPT-cAMP or TNFα (Fig. 2). In starved cells, both endogenous PKAc and p65 were immunoprecipitated by Myc-AKIP 1A (lane 4), but not a control Myc vector alone (lane 1). Consistent with previous observations (Fig. 1*A*), stimulation of cells with 8-CPT-cAMP resulted in a significant decrease in PKAc binding to AKIP 1A (lane 5, top panel), but the interaction of p65 with AKIP 1A was not significantly altered (lane 5, second panel). Similar to previously published results, stimulation with TNFα had no significant effect on the interaction of AKIP1 with either PKAc or p65 (lane 6, top two panels) (34). In other experiments, stimulation of cells with PMA did not alter interaction of AKIP 1A with PKAc or p65 (data not shown). In the presence of vector alone, neither the PKAc nor p65 was pulled down in resting or stimulated cells (lanes 1–3).

**Figure 3 pone-0018713-g003:**
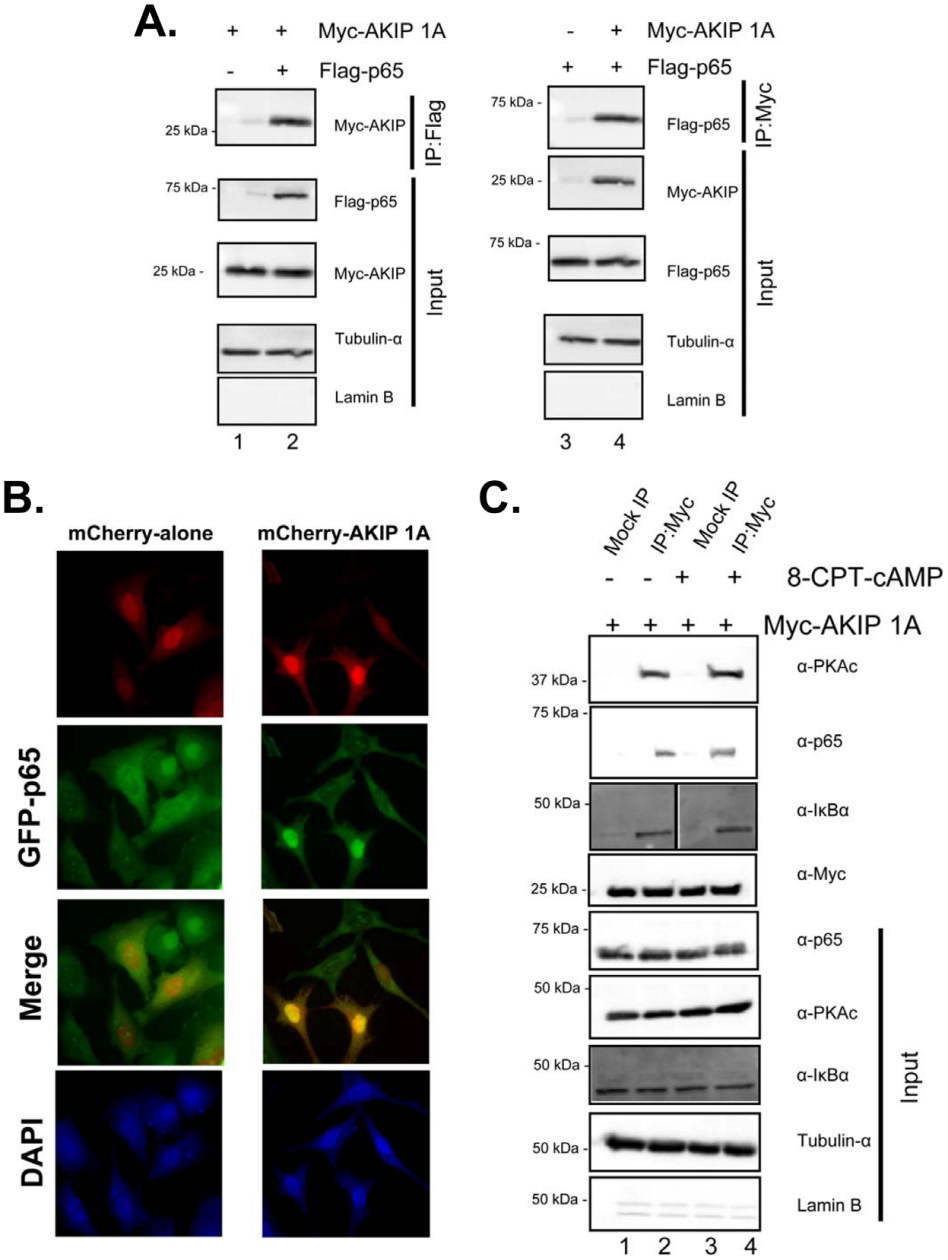
The p65 subunit of NF-κB binds AKIP 1A in the cytosol of unstimulated HEK 293 cells. A) *(left)* HEK 293 cells were transfected with Myc-AKIP 1A in the presence or absence of Flag-p65 WT. Cells were fractionated as described in the Methods section to yield a cytosolic fraction and Myc-AKIP 1A was immunoprecipitated and separated by SDS-polyacrylamide gels followed by Western blotting for Flag-p65. *(right)* HEK 293 cells were transfected with Flag-p65 WT in the presence or absence of Myc-AKIP 1A and fractionated as described above. Samples were Western blotted for Myc-AKIP 1A. Both fractions were free from nuclear contamination based on blotting of cytosolic fractions for the cytosolic marker tubulin-α, but not the nuclear marker lamin B. Data are representative of three replicate experiments. B) HeLa cells were transiently transfected with GFP-p65 in the presence of mCherry alone, or mCherry AKIP 1A. After 30 hours, cells were serum starved overnight, fixed, and imaged as described in Methods and Materials. C) Western blots of cytosolic fractions from immunoprecipitated Myc-AKIP 1A for PKAc, p65, and IκBα. Cytosol fractions were Western blotted for soluble p65, PKAc, and IκBα as well as the tubulin (cytosolic marker) and lamin B (nuclear marker). Data shown is with Myc-AKIP 1A in the absence (lanes 1 and 2) or presence of 8-CPT-cAMP (50 µM).

Once it was determined that AKIP 1A, PKAc, and p65 interact in resting or serum starved cells, we next wanted to determine whether the interaction was formed in the cytosol or nucleus. Both Myc-AKIP 1A and Flag-p65 interacted in the cytosol of resting cells (Fig. 3*A*, lanes 2 and 4), while control immunoprecipitations did not (Fig. 3*A*, lanes 1 and 3). The presence and purity of the cytosolic fraction was by determined using the cytosolic marker tubulin-α and the nuclear marker, Lamin B. Epifluorescence microscopy was employed to further explore whether p65 and AKIP 1A co-localize in the cytosol of unstimulated cells. A merged image of mCherry AKIP and GFP-p65 indicated that the two proteins were found at similar cytosolic and nuclear locations when compared with a diffusely localized mCherry vector alone (Fig. 3*B*). To determine whether the complex between AKIP 1A, PKAc, and p65 formed in the cytosol, overexpressed Myc-AKIP 1A was immunoprecipitated from cytosolic fractions of HeLa cells (Fig. 3*C*). In unstimulated and 8-CPT-cAMP stimulated cells both PKAc and p65 were associated with Myc-AKIP 1A (lanes 2 and 4, top panels). Control immunoprecipitations did not significantly bind endogenous PKAc or p65 (lanes 1 and 3). To determine whether the PKAc/AKIP 1A/p65 complex was a distinct complex from the p65/p50/IκBα complex, we also tested whether IκBα was in the complex with AKIP 1A immunoprecipitation. IκBα was present in the complex, suggesting that the AKIP 1A bound pool of p65 overlaps with the larger p65/p50/IκBα complex (panel 3). The observation that PKAc is associated with Myc-AKIP 1A in the cytosol of 8-CPT-cAMP stimulated cells indicates that the dissociation of PKAc from AKIP1A occurs after translocation into the nucleus (see Discussion).

### Defining primary binding region of AKIP 1A on PKAc

**Figure 4:**
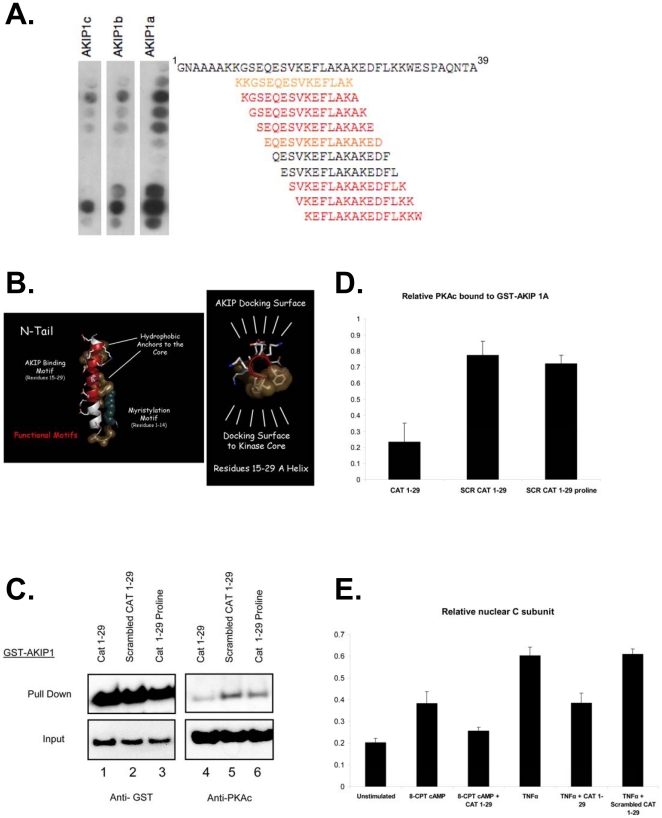
Defining sites of interaction on PKAc with AKIP 1A. A) Fifteen-residue peptides covering the catalytic domain (amino acids 1-38) of PKAc were staggered by one amino acid, spotted onto a membrane, and probed with AKIP1 splice variants proteins. Binding was detected with anti-AKIP1 antibody. The blot showed differential binding to the splice variants with the most intense binding to AKIP 1A, the full length protein. The residues in orange are weak binding and in red the strongest binding peptides. B) The amino terminal helix of PKAc is highlighted with exposed amino acids facing out towards the AKIP 1A binding surface: these amino acids may be important for the interaction with AKIP 1. C) HEK 293 cells were transfected with GST-AKIP 1A in the presence of the 1-29 PKAc disruptor peptide. Scrambled 1-29 peptide and a proline mutant at positions 20 and 22 to disrupt subsequent integrity of 1-29 helical structure served as controls. After 24 h, cells harvested and subjected to GST pull downs and immunoblotting showed that AKIP1/PKAc interaction was disrupted by the 1-29 peptide but not the two controls. D) Quantification of the amount of the relative amount of PKAc bound to GST-AKIP 1A. E) HeLa cells were transfected with Myc-AKIP 1A in the presence or absence of either CAT 1-29 or scrambled CAT 1-29. Cells were stimulated with 8-CPT-cAMP (50 µM) or TNFα (1 ng/ml) for 1 hour, fractionated to obtain nuclear enriched fractions and nuclear PKAc levels were measured by Western blot and quantified.

PKAc preferentially interacts with the AKIP 1A isoform, and the PKAc binding site was previously mapped to the amino terminus. To further map the interaction site(s) on PKAc, we performed a peptide walk to determine specific amino acids required for PKAc to bind to the three AKIP1 splice variants. Specifically, 15-mer peptides derived from the amino terminal 38 amino acids of PKAc were spotted onto a membrane and overlaid with *in vitro* translated AKIP 1A, AKIP 1B, or AKIP 1C proteins as previously described [11]. The three AKIP1 isoforms specifically bound clusters of sequences, shown in Fig. 4*A*, with the sequence of the spotted peptide shown to the right of the peptide spot. The complete peptide walk of AKIP 1A over the amino terminus of PKAc and the identified binding sites are shown in Figure S1. Exposed lysines at either end of the peptide appear to enhance AKIP1 binding to PKAc. To further define the optimal binding regions and critical amino acids within this region, peptides containing amino acids 11–30 were systematically shortened by one amino acid from the amino and/or carboxy terminal and tested for AKIP 1A binding (Figure S2). A peptide composed of amino acids 15–29 was identified as a minimal binding region. Figure 4*B* indicates the exposed amino acids on the solvent exposed surface of the N-terminal helix of PKAc that are thought to be important for interaction with AKIP 1A.

Previously, AKIP1 was found to enhance translocation of PKAc into the nucleus of HeLa cells stimulated with forskolin [11]. To determine whether this minimal peptide could disrupt the interaction of PKAc and AKIP 1A, HA-tagged 1-29 PKAc peptide (CAT 1-29) was co-transfected into HeLa cells with GST-AKIP 1A (Fig. 4*C*). Cells transfected with CAT 1-29 and GST-AKIP 1A had a significantly reduced capacity to pull down endogenous PKAc (lane 4). However, transfection with either a scrambled CAT 1-29 (SCR CAT 1-29; lane 5) or a 1-29 peptide in which two prolines were added to disrupt the alpha helical structure (CAT 1-29 proline; lane 6) did not interfere with PKAc binding to GST-AKIP 1A. In mock immunoprecipitated cells, interaction of GST-AKIP1A and endogenous PKAc was similar to the scrambled peptide (data not shown). Quantification of the interaction of endogenous PKAc with GST-AKIP 1A in the presence of CAT 1-29, scrambled CAT 1-29, and CAT 1-29 proline is shown in Fig. 4*D*, illustrating a decrease in binding using the CAT 1-29 disruptor peptide compared to the controls. Next, we tested whether transfection of cells with Myc-AKIP 1A and the wild-type CAT 1-29 disrupting peptide could block nuclear import of endogenous PKAc and provide evidence for a biological function (Fig. 4*E*). Nuclear PKAc from fractionated HeLa cells, as determined by the presence of lamin B, was measured (data not shown). In agreement with previous reports [34], PKAc redistributed to the nucleus upon stimulation with either 8-CPT cAMP or TNFα. Translocation was partially blocked upon addition of CAT 1-29 either in the presence of 8-CPT cAMP or TNFα, but not with scrambled CAT 1-29. Taken together, these results suggest that a peptide encompassing the first 29 amino acids of PKAc effectively disrupts the AKIP 1A-PKAc interaction and decreases nuclear import of PKAc.

### Defining primary binding region of PKAc and p65 on AKIP 1A

**Figure 5:**
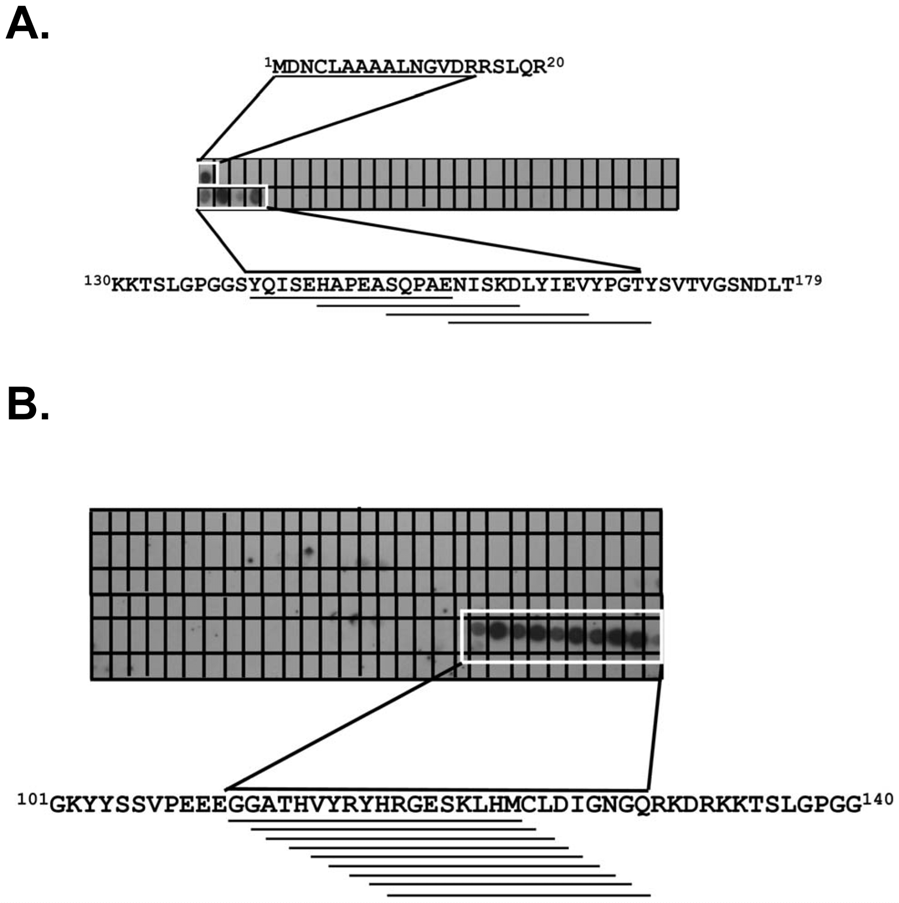
Identification of PKAc and p65 interaction sties on AKIP 1A by peptide array analysis. A peptide overlay of AKIP 1A with the purified catalytic domain of PKAc and p65. Fifteen-residue peptides covering amino acids 1-210 of AKIP 1A staggered by five amino acids (A) or eighteen-residue peptides covering amino acids 1-210 of AKIP 1A staggered by one amino acid (B) were spotted onto a membrane and overlaid with recombinant PKAc (A) or *in vitro* translated p65 (B). Binding of PKAc and p65 binding were detected by Western blotting using antibodies to PKAc or p65. The sequence, shown above or below the data, highlights the residues required for the interaction.

Our studies demonstrate that both PKAc and p65 directly bind AKIP 1A and immunoprecipitation experiments indicate that this complex exists in the cytosol of serum starved cells. Therefore, using peptide blot analysis, we next wanted to determine whether the binding sites of PKAc and p65 on AKIP 1A were similar or distinct (Fig. 5*A* and *B*). To identify PKAc binding sites, 15-mer peptides encompassing the entire AKIP 1A protein were spotted onto a membrane and incubated with recombinant purified PKAc. PKAc bound two specific clusters of amino acids, the first in exon 1 at the amino terminus of AKIP 1A and the second in exons 5 and 6 (Fig. 5*A*). To identify p65 binding sites, 18-mer peptides encompassing the entire AKIP 1A protein were spotted onto a membrane and incubated with *in vitro* translated p65 protein (Fig. 5*B*). The region of p65 binding was further narrowed to identify specific binding determinants (data not shown). The results indentified a binding site on AKIP 1A that did not overlap with the PKAc binding site. The amino acids required for p65 interaction with AKIP 1A encompassed a 12-mer peptide of GGATHVYRYHRG.

### Determining the rate of PKAc and AKIP1 mediated p65 translocation

**Figure 6:**
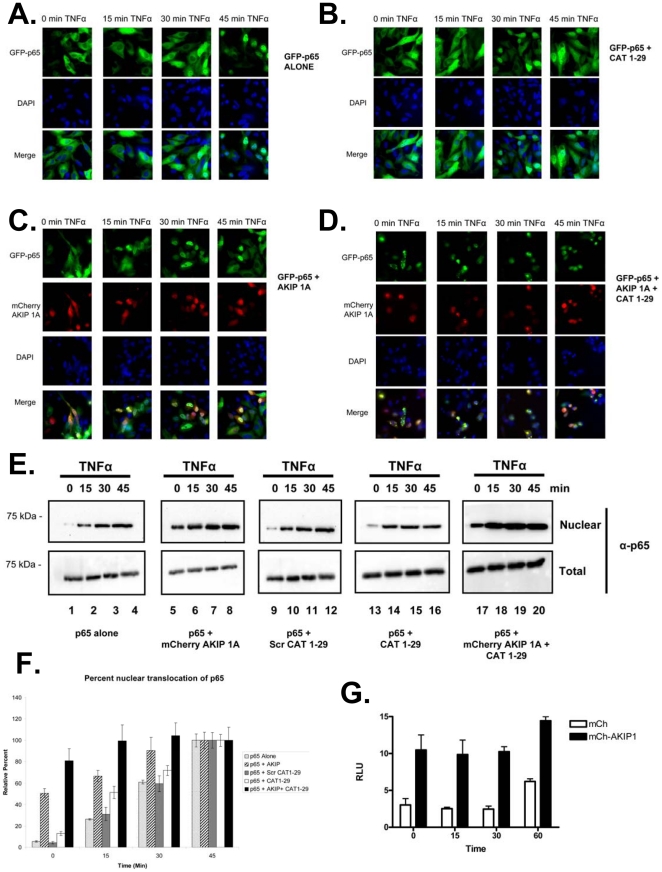
Blocking access of PKAc to p65 accelerates the kinetics of nuclear translocation of p65 in the presence of TNFα and augments p65 transcriptional activity. HeLa cells transfected with A) GFP-p65 WT alone, or with B) CAT 1-29 peptide, C) mCherry AKIP 1A, or D) both CAT 1-29 peptide and mCherry AKIP 1A. Cells were stimulated with TNFα (1 ng/ml) for 0, 15, 30 or 45 min, then fixed and permeabilized. Data were collected as described in Methods and Materials. DAPI staining of the nucleus and merged images are shown for each of the conditions in lower panels. E) HEK 293 cells transfected with either mCherry AKIP 1A, scrambled CAT 1-29 peptide, CAT 1-29 peptide, or mCherry AKIP 1A and CAT 1-29 peptide were stimulated with TNFα for 0, 15, 30, or 45 min. Representative Western blots demonstrate the accumulation of endogenous p65 in the nucleus compared to total p65 levels. F) Quantification of nuclear p65 levels. The amount of nuclear p65 was calculated based on normalized values of total p65 protein. G) HeLa cells were transfected with a NF-κB luciferase reporter in the presence of either mCherry alone or mCherry AKIP 1A and stimulated for up to 1 h with TNFα (1 ng/ml). Increased basal and TNFα stimulated p65 transcriptional activity was observed in the presence of AKIP 1A.

Once the binding regions of AKIP 1A with PKAc and p65 had been identified, we next wanted to determine whether disruption of the complex played a functional role in cells. First, we tested whether disrupting the interaction had an effect on the rate of nuclear accumulation of p65 in response to TNFα. HeLa cells were transfected with GFP-p65 alone (Fig. 6*A*), or in the presence of either CAT 1-29 peptide (Fig. 6*B*), mCherry AKIP 1A (Fig. 6*C*), or with both CAT 1-29 peptide and mCherry AKIP 1A (Fig. 6*D*). Cells were stimulated for up to 45 minutes with TNFα and nuclear accumulation of GFP-p65 over the course of the experiments was measured by epifluorescence microscopy. Over the TNFα time course, GFP-p65 translocated to the nucleus at a rate comparable to what others have observed [35]. Disruption of the interaction between PKAc and AKIP 1A by expression of the CAT 1-29 accelerated nuclear accumulation of GFP-p65 (compare Fig. 6*A* and Fig. 6*B*, 30 min time points). A more dramatic effect on the nuclear accumulation of GFP-p65 was observed when mCherry AKIP 1A alone was over expressed (Fig. 6*C*; see Discussion). Within 15 minutes of TNFα stimulation, a majority of the GFP-p65 had accumulated in the nucleus of HeLa cells overexpressing mCherry AKIP 1A. Double expression of CAT 1-29 and mCherry AKIP 1A resulted in nuclear accumulation of GFP-p65 in the absence of stimulus (Fig. 6*D*). To ensure that translocation of endogenous p65 was similar to GFP-p65, biochemical fractionation of HeLa cell lysate was performed on untransfected cells (Fig. 6*E*, lanes 1–4), cells transfected with mCherry AKIP 1A (lanes 5–8), scrambled CAT 1-29 (lanes 9–12), CAT 1-29 (lanes 13–16), or cells transfected with both mCherry AKIP 1A and CAT 1-29 (lanes 17–20). In untransfected cells, endogenous p65 was primarily excluded from the nucleus in starved cells in the absence of stimulus and gradually accumulated in the nuclear fraction during the 45 minute stimulation with TNFα. Cells transfected with either mCherry AKIP 1A (lanes 5–8) or CAT 1-29 (lanes 13–16) both displayed enhanced nuclear translocation of p65 in response to TNFα, but translocation of p65 in cells transfected with scrambled CAT 1-29 did not change significantly compared with untransfected cells (Fig. 6*F*). As with the GFP-p65, the double transfection of mCherry AKIP 1A and CAT 1-29 resulted in a substantial increase in the amount of p65 found in the nucleus in the absence of TNFα when compared with the total amount of p65 detected from whole cell lysates from the same samples (lanes 17–20). The relative amount of nuclear p65 compared with total p65 under each of these conditions from multiple experiments is plotted in Fig. 6*F*. The same effect of mCherry AKIP 1A and CAT 1-29 was observed when cells were stimulated with TNFα/8-CPT-cAMP (Figure S3). We next wanted to determine whether enhanced nuclear import of p65 in the presence of AKIP 1A was linked to an increase in transcriptional activity. HeLa cells were transfected with a luciferase gene reporter downstream of a NF-κB response element (Fig. 6*G*) and stimulated with TNFα for 1h. Enhanced luciferase activity was observed in all cells expressing mCherry AKIP 1A compared to control cells, whether serum starved or stimulated with TNFα.

**Figure 7:**
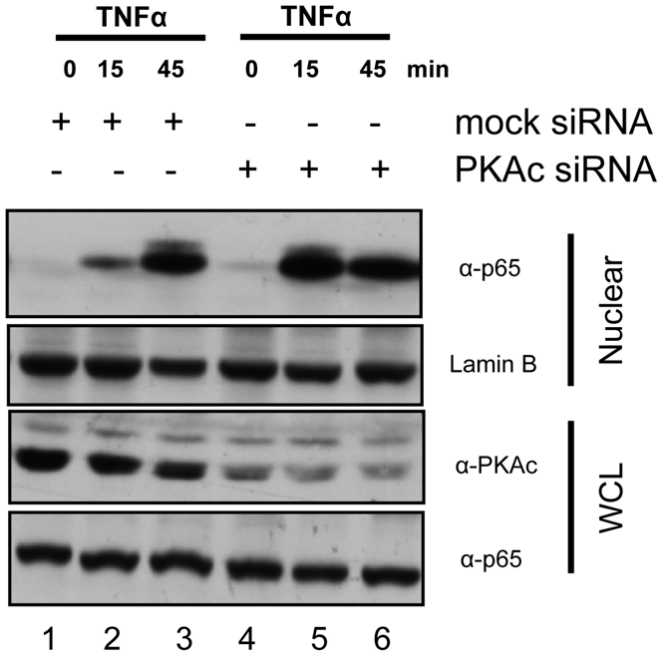
Knock down of endogenous PKAc accelerates nuclear accumulation of endogenous p65. HeLa cells were transfected with mock siRNA (lanes 1–3) or PKAc siRNA (lanes 4–6) for 36 hours, serum starved for 12 hours, and then stimulated with TNFα for 0, 15, or 45 min. Samples were analyzed on 10% SDS-polyacrylamide gels and Western blotted with antibodies to p65, PKAc, and lamin B.

A siRNA approach was employed to determine whether PKAc could directly regulate the rate of p65 nuclear translocation in response to TNFα (Fig. 7). PKAc (both α and β isoforms) was knocked down in HeLa cells and the rate of p65 nuclear translocation was measured as previously described. Cells with reduced PKAc protein displayed a more robust translocation of p65 to the nucleus in response to TNFα at 15 minutes compared with cells transfected with a scrambled siRNA (lanes 2 and 5). There was no difference in the amount of p65 in the nucleus in unstimulated cells (lanes 1 and 4) or cells after 45 min of treatment with TNFα (lanes 3 and 6).

**Figure 8:**
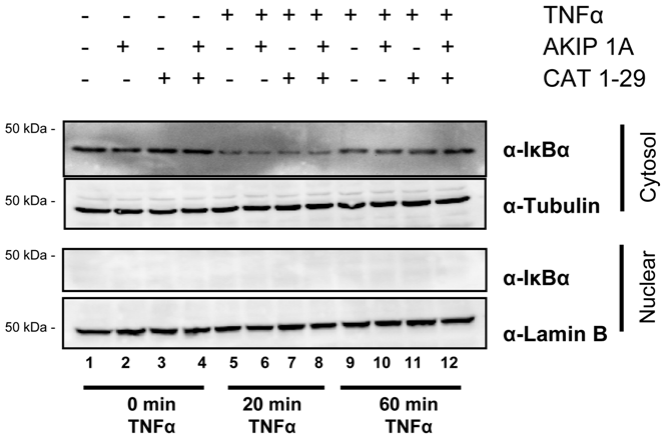
AKIP 1A and CAT 1-29 do not alter IκBα degradation rates after TNFα stimulation. HeLa cells were transfected with Myc-AKIP 1A in the presence or absence of HA-CAT 1-29 and stimulated with TNFα for 0, 20, or 60 min with TNFα. Representative Western are shown of samples analyzed on 10% SDS-polyacrylamide gels and Western blotted with antibodies to p65, PKAc, and lamin B.

Results from the siRNA experiments indicate that PKAc may regulate the rate at which p65 enters the nucleus. Given that AKIP 1A is limiting in cells, we hypothesized that only a small population of the PKAc and p65 exist in this complex. NF-κB is primarily retained in the cytosol by IκB isoforms that mask the NLS on p65 [17]. Upstream activation by a number of stimuli, including TNFα or PMA, results in phosphorylation of IκB and subsequent targeting and degradation of IκB by the proteosome. It is possible that the AKIP 1A, PKAc, p65 interaction we observe is in a larger complex with other p65 binding proteins. This suggests that AKIP 1A and PKAc may regulate something other than p65 directly. One logical thought was that AKIP 1A might influence the rate of IκB degradation. To test this, HEK 293 cells mock transfected or transfected with Myc-AKIP 1A were stimulated with TNFα for up to 60 minutes then fractionated into cytosolic and nuclear fractions. The Western blot in Fig. 8 (top panel) shows the rate of IκBα degradation in the cytosol after TNFα stimulation at 0, 20 and 60 minutes. Before addition of TNFα, cytosolic IκBα levels of untransfected cells were identical with those transfected with AKIP 1A and/or CAT 1-29 (lanes 1–4). After 20 min of TNFα stimulation, there was a decrease in the amount of IκBα protein in all samples with no concomitant increase in nuclear IκBα detected (lanes 5–8). Consistent with other observations [22], IκBα levels began to recover after 60 min (lanes 9–12). The presence of AKIP 1A and/or CAT 1-29 did not significantly alter the rate of IκBα degradation or synthesis, indicating that co-localization of AKIP 1A and p65 does not interfere with canonical NF-κB signal transduction.

**Figure 9:**
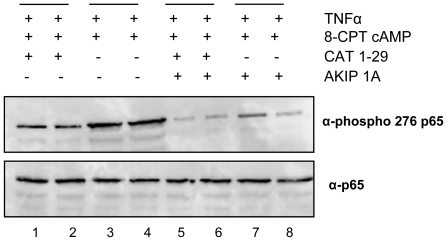
Disruption of the interaction between AKIP 1A and PKAc decreases phosphorylation of p65 at serine 276 in the cytosol. HeLa cells were transfected with CAT 1-29 (lanes 1 and 2), AKIP 1A (lanes 7 and 8), or CAT 1-29 and AKIP 1A (lanes 5 and 6) in the absence (lanes 1, 3, 5, 7) or presence (lanes 2, 4, 6, 8) of TNFα/8-CPT-cAMP. Samples were fractionated and the cytosolic fraction analyzed on 10% SDS-polyacrylamide gels and Western blotted with antibodies to phospho-Ser 276 p65 and p65. Lines indicate duplicate samples.

Recently, Dong *et al.* found that mutation of the PKAc phosphorylation site on p65 at position 276 to an alanine was embryonic lethal [32]. Death in this system was attributed to epigenetic repression through recruitment of histone deacetylases to transcription hot-spots in the nucleus. Based on this information, it was possible that AKIP1A was acting as an adaptor protein that brings PKAc into the proximity of p65 and facilitates phosphorylation. To test whether phosphorylation of Ser 276 could be modulated by AKIP1A, we over-expressed AKIP1A and/or CAT 1-29 in HeLa cells. After a 45 minute incubation with TNFα/8CPT-cAMP (lanes 2, 4, 6, 8), phosphorylation at serine 276 in the cytosol was measured by Western blotting and compared with serum starved cells (lanes 1, 3, 5, 7) (Figure 9). In untransfected cells (lanes 3 and 4), phospho-276 levels were high in both starved and stimulated conditions. Transfection with CAT 1-29 (lanes 1 and 2), AKIP 1A (lanes 7 and 8), and CAT 1-29/AKIP 1A (lanes 5 and 6) dramatically lowered the amount of phosphor-276 observed in the cytosolic fraction of cells.

**Figure 10:**
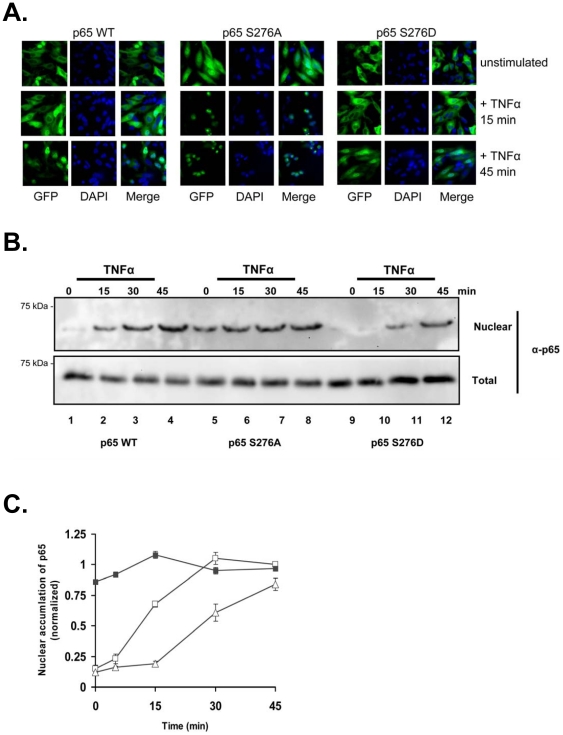
Replacement of serine 276 on p65 with an alanine accelerates nuclear translocation, while replacement with an aspartic acid slows nuclear translocation. A) HeLa cells transfected with GFP-p65 WT, GFP-p65 S276A, or GFP-p65 S276D were stimulated with TNFα (1 ng/ml) for 0, 15, or 45 min. Cells were fixed and data were collected as described in the Methods section. B) HEK 293 cells were transfected with Flag-p65 WT (lanes 1–4), Flag-p65 S276A (lanes 5–8), or Flag-p65 S276D (lanes 9–12) and stimulated with TNFα (1 ng/ml) for 0, 15, 30, or 45 min. Representative Western blots demonstrate the accumulation of the different p65 constructs in the nucleus compared to total p65 levels. C) Quantification of nuclear p65 levels calculated based on normalized values of total p65 protein. Legend: p65 alone (□), p65 S276A (▪), p65 S276D (Δ).

Next, we wanted to determine whether mutation of serine 276 could mimic the nuclear translocation of p65 observed in the presence of AKIP 1A and CAT 1-29. Serine 276 in p65 was mutated to alanine (S276A) to mimic the dephosphorylated p65 and aspartic acid (S276D) to mimic the phosphorylated p65. HeLa cells were transfected with each of the GFP-tagged constructs and stimulated with TNFα for up to 45 minutes (Fig. 10*A*). Wild-type p65 translocated to the nucleus with kinetics observed previously (left panels). The S276A mutant rapidly accumulated in the nucleus of cells in the absence of stimulus (middle panels). Conversely, the S276D mutant displayed slightly delayed translocation of p65 into the nucleus (right hand panels). Biochemical fractionation of cytosolic and nuclear proteins from HeLa cells transfected with Flag-tagged versions of the p65 constructs was performed to quantify translocation (Fig. 10 *B* and *C*). More Flag-p65 WT was observed in the nuclear fractions at 0, 15, 30 minutes than with endogenous p65 (lanes 1, 2, and 3) which may be an artifact of overexpression. However, under the same conditions, there was substantially more p65 S276A observed in the nucleus of unstimulated cells compared to wild-type p65 (lanes 5–8). Conversely, nuclear accumulation of p65 was slowed in the S276D mutant (lanes 9–12).

## Discussion

PKAc has been shown to trigger the activation and translocation of the p65 component of NF-κB. However, the mechanism of translocation is unknown and was believed to be energy dependent [33]. Furthermore, though the authors showed several years ago that phosphorylation by PKAc was a key event in p65 activation and translocation, the substrate for this reaction is still unknown [33]. Our data shows that AKIP1 is the missing link regulating both translocation and activation. We first demonstrate that AKIP 1A, the catalytic subunit of PKA, and the p65 subunit of NF-κB can form a cytosolic complex. This complex could be modulated through activators of PKA, overexpression of AKIP 1A, and over-expression of a peptide (CAT 1-29) that blocks the interaction of PKAc and AKIP 1A. Second, we show that both PKAc and AKIP1 regulate the rate at which NF-κB translocates into the nucleus in response to stimulus. TNFα mediated translocation of p65 into the nucleus of HeLa cells was greatly enhanced either by siRNA knock down of PKAc or over-expression of AKIP1. Third, we provide further evidence showing that the phosphorylation of p65 by PKAc is essential in its translocation. Expression of either AKIP 1A or concomitantly with CAT 1-29 resulted in a constitutive localization of p65 in the nucleus, indicating that AKIP1 regulates the rate at which p65 enters the nucleus (Figs. 6D and 6E). There was, however, no effect of AKIP 1A or CAT 1-29 on the rate of IκB degradation (Fig. 8), suggesting the canonical NF-κB nuclear translocation pathway remained intact. IκBα was demonstrated to be present in this complex (Fig. 3C), suggesting that the pool of AKIP1 bound to PKAc and p65 overlaps with the total population of cytosolic NF-κB.

Based on mRNA levels and protein expression data, the levels of endogenous AKIP 1A are significantly lower than p65 or PKAc. The observation that AKIP1 isoforms are limiting in the complex formation suggests that under normal cellular conditions, every AKIP1 protein will be associated with both a PKAc and p65. Therefore, when AKIP1 is no longer limiting, p65 nuclear translocation is altered. Each one of these salient features is addressed in detail below.

### Isoform specificity dictates AKIP1 interactions

The two other groups that worked on AKIP1 showed either the enhancement or abrogation of the transcriptional activity of p65 upon binding AKIP1. Though both groups have used similar stimuli and cell lines, they obtained opposite results especially with respect to the effects on the endogenous protein. Our studies found that the cell lines used had very little AKIP1 (data not shown). In human cell lines, the problem is further compounded by the presence of splice variants and thus the results obtained could be misleading. To clarify this problem, we systematically studied isoform specificity of AKIP1 in multiple cell lines and found that the predominant endogenous AKIP1 isoform present in the cells was AKIP 1B (data not shown). Subsequently, we used biochemical and peptide array technology to provide insight into the function of the various AKIP1 isoforms and the interactions of PKAc and p65. Biochemical data and peptide blot analysis provide evidence that the 1A isoform of AKIP preferentially binds PKAc (Fig. 1A and Fig. 4A). Binding to the N-terminus of PKAc was similar to that of the full-length protein (compare Fig. 1A to [Fig pone-0018713-g002] [Fig pone-0018713-g003] 4A). One interacting region was detected in the array, amino acids 8-30 (KKGSEQESVKEFLAKAKEDFLKKW). This region of PKAc is within the bait region originally used to identify AKIP1. Peptide arrays further identified a major binding determinant for PKAc was located in exons 5 and 6 of AKIP 1A (Fig. 5A). This region has a putative PKAc phosphorylation site and thus provides rationale for the increased binding observed between PKAc and AKIP 1A. Though AKIP 1B also has these sites, the lack of exon 3 may result in differential folding which could explain decreased binding. Mapping studies of p65 binding sites on AKIP 1A point to a conserved small region within exon 4 as the predominant binding site (Fig. 5B). This region is common to all three AKIP1 isoforms, suggesting p65 might interact with all AKIP1 splice variants. Evidence for p65 binding to multiple AKIP1 isoforms has been demonstrated in two different studies [34], [35]. These results indicate that PKAc and p65 bind different regions of AKIP 1A allowing them to form the complex. Furthermore, selective disruption of the PKAc interaction through the CAT 1-29 peptide does not affect the binding of p65 to AKIP 1A (data not shown). These data have implications for the functions of the individual isoforms of AKIP1. Recently, the role of AKIP1 in p65 mediated gene expression has been studied. Subsequently, the AKIP 1A isoform was identified as an enhancer of NF-κB dependent transcription [34]. The difference between these effects was postulated to be a result of agonist concentration, but may be related to differential PKAc binding to the AKIP1 isoforms, rate of p65 nuclear accumulation, or access of p65 to other binding partners.

### Stimulus and spatial localization regulate PKAc interaction with AKIP1

Complicating the isoform-specific interactions of AKIP1 with known binding partners is that elevation of cAMP accelerates dissociation of PKAc from AKIP1. In whole cell lysate, stimulation with 8-CPT cAMP results in a net dissociation of PKAc from AKIP1 (Fig. 1A). This appears to be stimulus dependent because no decrease in the interaction between PKAc and AKIP 1A was observed in the presence of TNFα. Under all conditions such as resting, serum starvation, or upon direct stimuli, the interaction between AKIP1 and p65 remains constant. Given the interaction of PKAc and AKIP1 in both the cytosolic and nuclear fractions of cells, dissociation of PKAc from AKIP1 was further explored after fractionation of cells. In the cytosol, 8-CPT cAMP there was no change in the interaction (Fig. 3E). However, there was increase in the nuclear accumalation of PKAc in the presence of AKIP1 and increased transcription (Fig. 4E, 1B). However, we were unable to establish either interaction or co-localization between PKAc and AKIP1 in the nucleus either in the presence of 8-CPT cAMP or TNFα (data not shown). We hypothesize that over-expression of AKIP1 uncouples the PKAc and p65 interaction. This is based on the change in relative nuclear accumulation of PKAc and p65 in the presence of AKIP1. In Fig. 6E, both the rate and total p65 imported into the nucleus is increased in the presence of AKIP 1A and CAT 1-29. Conversely, the amount of PKAc in the nucleus is reduced under the same conditions though the amount of PKAc in the nucleus remained unchanged in the presence of AKIP 1A alone. Separately, we observe that AKIP1 dependent-PKAc transcription is increased in the presence of stimulus where PKAc is no longer associated with AKIP 1A (Fig. 1B). Taken together, these data suggest that AKIP 1A acts to shuttle PKAc and p65 into the nucleus, but that PKAc is rapidly dissociated from the complex once inside.

### Regulation of the rate of NF-κB translocation upon phosphorylation

**Figure 11:**
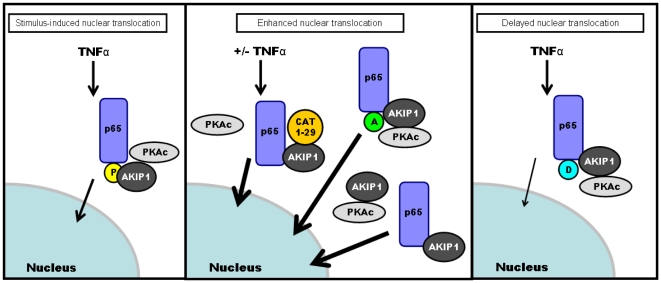
Model for PKAc-mediated enhanced p65 nuclear translocation. Translocation of p65 to the nucleus is regulated by external stimuli and canonical TNFα stimulation. Accelerated nuclear accumulation p65 is observed by disrupting the interaction between PKAc and AKIP, overexpressing the limiting AKIP, or by mutation of the PKAc phosphorylation site at serine 276 to alanine. Nuclear accumulation of p65 can be delayed by replacing serine 276 with a phosphomimetic.

Though NF-κB has been shown to be a substrate of PKA, the downstream effects of it on translocation and transcription are still largely an enigma. One paper suggested that the level of AKIP1 plays a determining role in either the activation or inhibition of p65 resulting in cell proliferation or cell death in cancer cell lines [36]. We show that AKIP1 is important also in regulating the rate of p65 into the nucleus. We next investigated whether this is a consequence of serine 276 phosphorylation or through some other mechanism. Phosphorylation of serine 276 on p65, a well described PKAc site, in cytosolic fractions was dramatically reduced in TNFα stimulated cells expressing AKIP 1A and/or CAT 1-29, suggesting that phosphorylation of p65 by PKAc regulated nuclear import. Mutation of serine 276 to alanine resulted in strong, constitutive nuclear localization of p65, while mutation to the phosphomimetic aspartic acid displayed reduced nuclear translocation kinetics (Fig. 10A and B). Thus, AKIP 1A serves as a scaffold that allows PKAc proximal contact with p65 in the cytosol and protects the serine 276 site from being phosphorylated. Work from Dong, *et al.* described the results from a knock-in mouse expressing NF-κB S276A mutant that were embryonic lethal [32]. In the wild-type animals, p65 phosphorylated at serine 276 could recruit CBP/p300 to specific transcription sites, however, when serine 276 was mutated to alanine, HDAC3 replaced CBP/p300 binding on p65. The lack of recruitment alone was insufficient to explain the lethality. The presence of HDAC3 acts by epigenetic repression of genes proximal to NF-κB sites. Thus, given the importance of serine 276 phosphorylation in the nucleus, we identified AKIP1 as not only an important regulator of nuclear translocation of p65 by positioning PKAc in proximity of serine 276 but also important in retaining both p65 and PKAc and enhancing their transcription. We postulate that this could be through AKIP1 shielding the phosphorylation site of p65 in the cytosol from PKAc, enhancing the translocation to the nucleus, wherein we believe PKAc phosphorylates p65 and is released from the complex allowing for the recruitment of CBP/p300 (Fig. 11). One aspect that has to be delved into is the mechanism of action of the various isoforms of AKIP1 in recruiting CBP/p300 or HDACs. These studies would lay a foundation for determining the role of AKIP1 in PKA mediated NF-kB transcription and in turn lead to a better understanding of cell proliferation and differentiation.

## Materials and Methods

### Reagents

The monoclonal antibody to PKAc was purchased from BD Biosciences (San Jose, CA, USA). An antibody to the Flag epitope was purchased from Sigma (St Louis, MO, USA). Antibodies to p65, phospho-NF-κB p65 (Ser 276), IκBα, and GST were purchased from Cell Signaling (Danvers, MA, USA). Antibodies to actin, tubulin, and lamin B were purchased from Santa Cruz Biotechnology (Santa Cruz, CA, USA). GFP- NF-κB p65 was a generous gift from Dr. Mel Simon (UCSD). Control siRNA and siRNA to PKAc was purchased from Santa Cruz Biotechnology. TNFα was purchased from Roche (San Francisco, CA, USA) and 8CPT-cAMP was purchased from Sigma Aldrich (St. Louis, MO). All other materials were reagent grade.

### cDNA constructs and Plasmids

Human AKIP 1A, AKIP 1B, and AKIP 1C were cloned into the pEBG vector containing an amino-terminal GST epitope tag. Myc AKIP 1A was generated by cloning into the pCMV Myc vector using Nco1 and Kpn1 restriction endonuclease sites. mCherry AKIP 1A was generated by cloning into the pCDNA3 mCherry vector using HindIII and Xho1 restriction endonuclease sites. HA-CAT 1-29 was generated by PCR amplification using the following primers: 5′ TAACAATGTACCCATACGATGTTCCGGATTACGCTatgggcaacgccgccgcc and 3′ ggagagcgtgaaagagttcctagccaaagccaaggaagatttcctgaaaaaatgaGCGGCCGCgat cctctcgcactttctcaaggatcggtttcggttccttctaaaggacttttttactcgccggcgctag CCCGGGCCCGCGGTACCGTCGACTGCAGAATTAtcattttttcaggaaatc. HA-CAT Scrambled was generated by PCR amplification using the following primers: 5′ TAACAATGTACCCATACGATGTTCCGGATTACGCTatggccagcgagaaagag and 3′ ccctaaaaaaccaggccaagggcagcaagaaattcgaactggaggtgttcggcgcctga gggattttttggtccggttcccgtcgttctttaagcttgacctccacaagccgcggact CCCGGGCCCGCGGTACCGTCGACTGCAGAATTAtcaggcgccgaacacctc. Both products were cloned into the phCMV-Xi clone vector using homologous recombination.

### Cell Culture and Transfection

All cell lines used in these studies (HEK 293 and HeLa; ATCC CRL-1573 and CCL-2, respectively) were maintained in Dulbecco's modified Eagle's medium (DMEM, BioWhittiker, Basel, Switzerland) containing 10% heat-inactivated fetal bovine serum (Omega Scientific Inc., San Diego, CA, USA). For transfections, cells (seeded at 1.2×10^6^ cells/10-cm dish) were grown to 70% confluence and transfected (2 µg total plasmid DNA and 60 µl Effectene per 10 cm dish) according to the manufacturer's protocol (Qiagen, Valencia, CA, USA). Thirty hours post transfection, cells were transferred to serum-free media for 18 h, and stimulated with 50 µM 8CPT-cAMP and/or 1 ng/ml TNFα.

### Cell Fractionation

Cells were fractionated using the Qproteome cell compartment kit to generate cytosolic and nuclear pools as described by the manufacturer (Qiagen).

### Luciferase Assays

The PKA and NF-κB activity was determined using the Dual-Luciferase® Reporter (DLR™) Assay System (Promega) according to the manufacture's instructions. Briefly, HeLa cells were cultured in 24 well plates and the cells were transfected with FuGENE 6 transfection reagent (Roche). To determine PKA activity, cells were co-transfected with 125ng of pGL4 CRE (cAMP response element) Luc2, 25 ng of pRL (hRluc gene that expresses the Renilla luciferase as a internal control) together with 125 ng of mCherry AKIP1A or mCherry for 18 hours and then the cells were stimulated with 100 mM forsokolin/IBMX for 0, 30, 60 and 120 mins. Similarly, to monitor NF-kB activity, cells were co-transfected with 125 ng of pGL-3κB HIV-Luc, 25 ng of pRL along with 125 ng of mCherry AKIP1A or mCherry. The cells were stimulated with 10ng/ml of TNFα for 0, 15, 30 and 60 minutes. The cells were then harvested in 100 µl of passive lysis buffer and subjected to the DLR activity assay. The output of the luciferase assays was in relative light units (RLU). Promoter activity bar graphs were generated and statistical analysis of the data performed using MS Office Excel and the GraphPad Prism software (GraphPad Prism software Inc., US).

### GST Fusion Protein Pull-down Assay

HeLa cells were transiently transfected with GST-tagged AKIP 1A, AKIP 1B, or AKIP 1C. Transfected cells were lysed in buffer A (50 mm Na_2_HPO_4_, 1 mm sodium pyrophosphate, 20 mm NaF, 2 mm EDTA, 2 mm EGTA, 1% Triton X-100, 1 mm dithiothreitol, 200 µm benzamidine, 40 µg ml^−1^ leupeptin, 300 µmphenylmethylsulfonyl fluoride). The detergent-solublized cell lysates were incubated with glutathione-Sepharose overnight at 4°C. Beads were washed twice in buffer A and twice in buffer B (300 mm NaCl, 50 mm Na_2_HPO_4_, 1 mm sodium pyrophosphate, 20 mm NaF, 2 mm EDTA, 2 mm EGTA, 1% Triton X-100, 1 mm dithiothreitol, 200 µm benzamidine, 40 µg ml^−1^ leupeptin, 300 µmphenylmethylsulfonyl fluoride), and proteins bound to the glutathione-Sepharose beads were analyzed by SDS-PAGE and Western blot analysis using chemiluminescence and quantified with a CCD camera using a Fluoro-Chem Q bio-imaging system (Alpha Innotech, Santa Clara, CA, USA).

### AKIP1 Overlay of the Catalytic Domain of PKA

The catalytic domain of PKA was divided into 18-amino-acid peptides, with a single amino-acid shift, and synthesized using the INTAVIS MultiPep peptide synthesizer (INTAVIS Bioanalytical Instruments AG, Koeln, Germany), which spotted the peptides onto an AC-S01 type amino-PEGylated membrane (INTAVIS AG). Following activation with ethanol, the membrane was blocked, washed with PBS-T, and incubated overnight with 100 nM human AKIP1 protein. This protein was generated by using untagged pRSET construct of AKIP 1A that was in vitro translated using rabbit reticlulate lysate. After incubation with the AKIP 1A, the peptide array was analyzed by Western blot analysis with polyclonal rabbit AKIP1 antibody generated previously (11).

### Immunoprecipitation

Antibodies to transfected Myc and Flag tagged constructs were used to immunoprecipitate proteins from cell lysates. Detergent soluble cell lysates and the indicated specific antibodies were incubated in binding buffer (100 mM KCl, 150 mM NaCl, 3.5 mM MgCl_2_, 10 mM Pipes, pH 7.3, 1 mM DTT, and 1 mM PMSF) overnight at 4°C, followed by a 1 h incubation with bovine serum albumin (Sigma)-coated protein A/G-Sepharose beads (GE Healthcare, Piscataway, NJ, USA). Beads were pelleted by centrifugation, and the pellet was washed three times in binding buffer containing 1% Nonidet-40 and three times in binding buffer alone. Immunoprecipitated proteins were separated by SDS-PAGE, transferred to nitrocellulose, and detected by Western Blot analysis.

### Immunofluorescence

HeLa cells grown on sterile coverslips were transfected with various GFP-p65 constructs in the absence or presence of mCherry AKIP 1A and/or HA-CAT 1-39 at 40% confluency. After 24 hours, cells were fixed with 4% paraformaldehyde for 15 min, permeabilized with PBS containing 0.5% Triton X-100 for 5 min and washed three times with PBS. After several washes with PBS containing 0.1% Triton X-100, the samples were mounted with Vectashield Hardset media containing DAPI (Vector Laboratories, Burlingame, CA, USA) and images were acquired on a Zeiss Axiovert microscope (Carl Zeiss Microimaging, Inc., Thornwood, NY, USA) using a MicroMax digital camera (Roper-Princeton Instruments, Acton, MA, USA) controlled by MetaFluor software (Universal Imaging, Corp., Sunnyvale, CA, USA).

## Supporting Information

Figure S1 A peptide overlay of the catalytic domain of PKAc with purified AKIP 1A. Fifteen-residue peptides covering the catalytic domain (amino acids 1-38) of PKAc staggered by one amino acid were spotted onto a membrane and overlaid with in vitro translated AKIP 1A (Pierce, TNT kit), and AKIP 1A binding was detected with a polyclonal anti-AKIP antibody. The residues highlighted in orange are weak binding peptides, and the residues highlighted in red are the residues delineating the stronger binding peptides. The boxed peptides were the strongest binders.
(TIF)

Figure S2 A peptide overlay of AKIP 1A with the purified catalytic domain of PKAc. The strongest AKIP1 binding peptide on PKAc, identified in Figure 2A, was sequentially truncated from the amino and carboxy terminal ends, as well as from both ends simultaneously to define the minimal PKAc binding peptide region. Peptides were spotted onto a membrane and overlaid with purified PKAc and p65 and detected by Western blotting using anti-PKAc or p65 antibodies.
(TIF)

Figure S3 Graph of p65 nuclear translocation of p65 in the presence of TNF/8CPT. HeLa cells transfected with Cells were stimulated with TNFα (1 ng/ml) for 0, 15, 30 or 45 min, then fixed and imaged. Data shown is quantification of nuclear p65 levels based on normalized values of total p65 protein (n = 3). Legend: p65 alone (▪), p65 + AKIP 1A (▴), p65 + AKIP 1A + CAT 1-29 (○), and p65 + CAT 1-29 (□).
(TIF)
