# Supplementary figures and images for: The Rate of NF-κB Nuclear Translocation Is Regulated by PKA and A Kinase Interacting Protein 1
Source: PLoS One. 2011 Apr 27;6(4):e18713. doi: 10.1371/journal.pone.0018713 (PMC3083391; doi:10.1371/journal.pone.0018713)

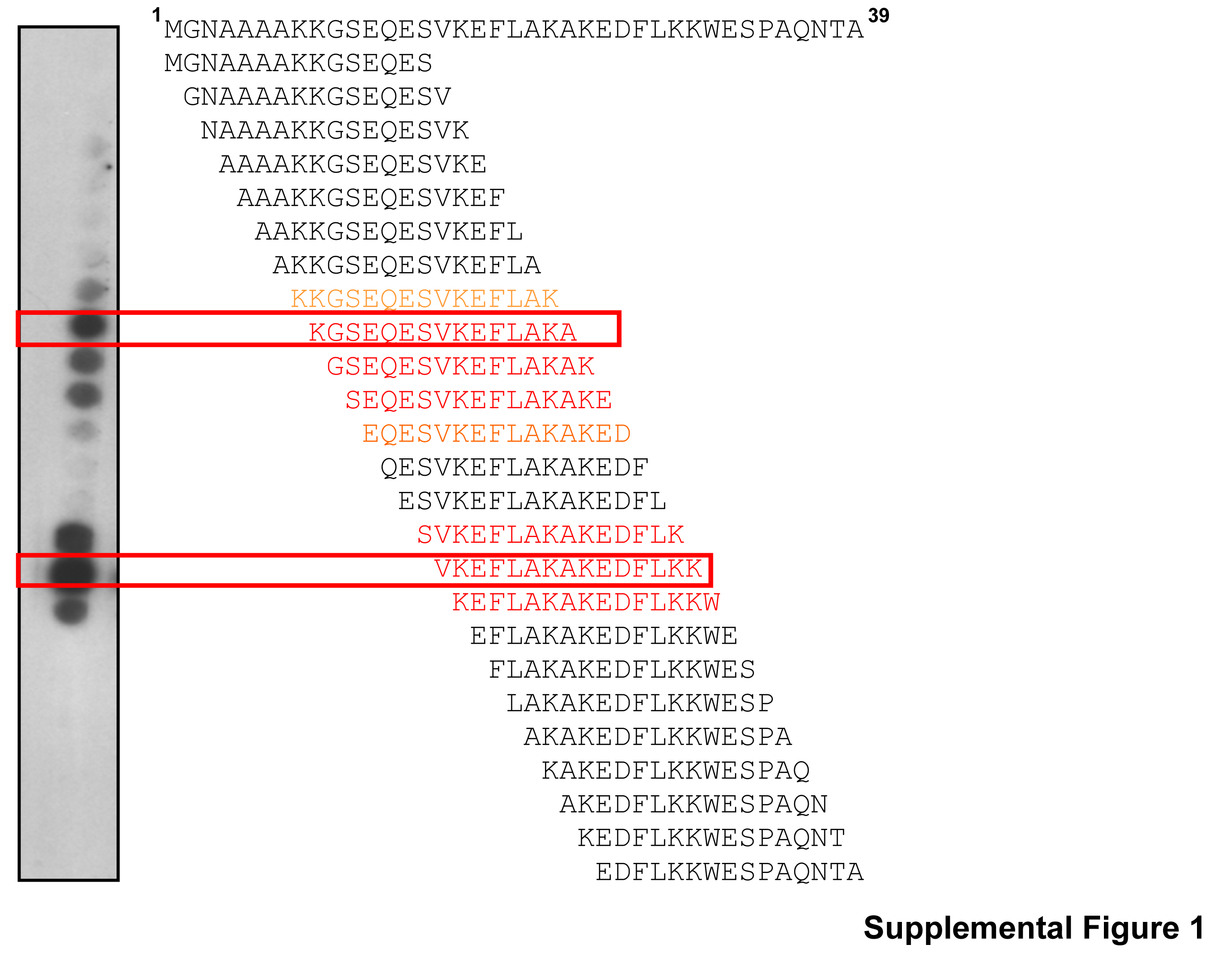

Supplement: Figure S1 — A peptide overlay of the catalytic domain of PKAc with purified AKIP 1A. Fifteen-residue peptides covering the catalytic domain (amino acids 1-38) of PKAc staggered by one amino acid were spotted onto a membrane and overlaid with in vitro translated AKIP 1A (Pierce, TNT kit), and AKIP 1A binding was detected with a polyclonal anti-AKIP antibody. The residues highlighted in orange are weak binding peptides, and the residues highlighted in red are the residues delineating the stronger binding peptides. The boxed peptides were the strongest binders. (TIF) [file pone.0018713.s001.tif]

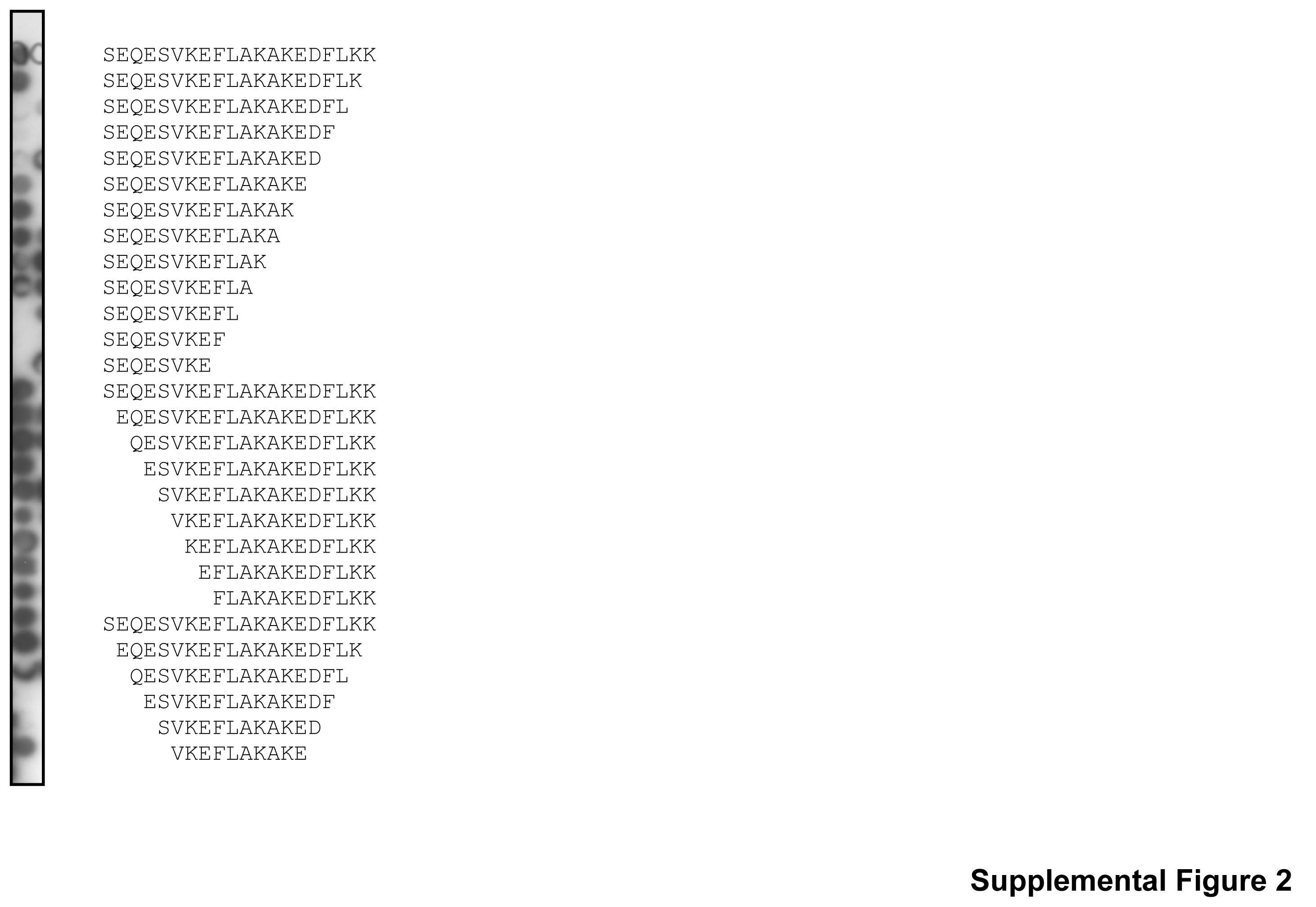

Supplement: Figure S2 — A peptide overlay of AKIP 1A with the purified catalytic domain of PKAc. The strongest AKIP1 binding peptide on PKAc, identified in Figure 2A, was sequentially truncated from the amino and carboxy terminal ends, as well as from both ends simultaneously to define the minimal PKAc binding peptide region. Peptides were spotted onto a membrane and overlaid with purified PKAc and p65 and detected by Western blotting using anti-PKAc or p65 antibodies. (TIF) [file pone.0018713.s002.tif]

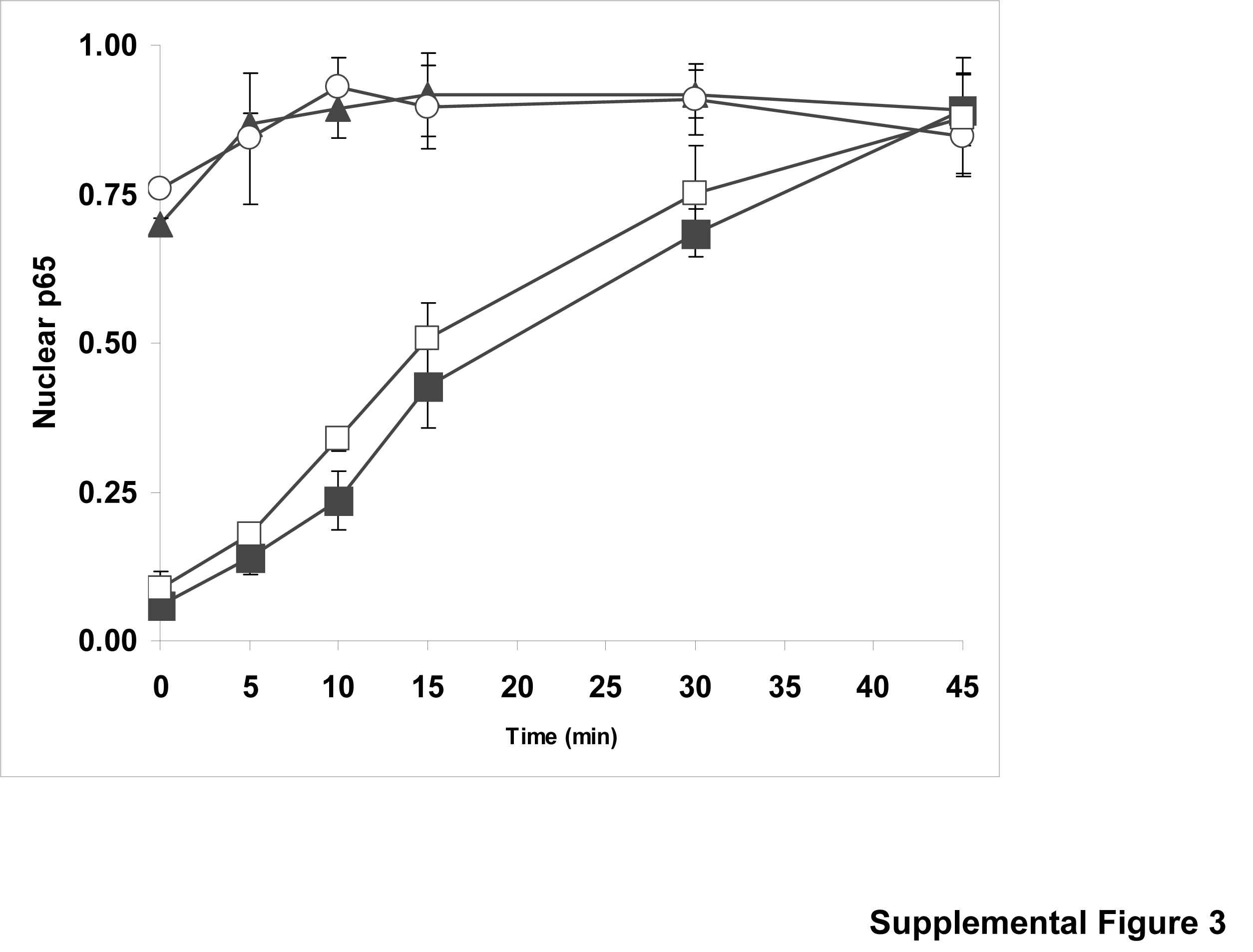

Supplement: Figure S3 — Graph of p65 nuclear translocation of p65 in the presence of TNF/8CPT. HeLa cells transfected with Cells were stimulated with TNFα (1 ng/ml) for 0, 15, 30 or 45 min, then fixed and imaged. Data shown is quantification of nuclear p65 levels based on normalized values of total p65 protein (n = 3). Legend: p65 alone (▪), p65 + AKIP 1A (▴), p65 + AKIP 1A + CAT 1-29 (○), and p65 + CAT 1-29 (□). (TIF) [file pone.0018713.s003.tif]
